# Revealing potential long non-coding RNA biomarkers in lung adenocarcinoma using long non-coding RNA-mediated competitive endogenous RNA network

**DOI:** 10.1590/1414-431X20176297

**Published:** 2017-08-07

**Authors:** T-G. Zhu, X. Xiao, Q. Wei, M. Yue, L-X. Zhang

**Affiliations:** 1 Department of Pulmonary Disease, The Affiliated Hospital of Changchun University of Chinese Medicine, Changchun, Jilin Province, China; 2 Department of Heart Disease, The Affiliated Hospital of Changchun University of Chinese Medicine, Changchun, Jilin Province, China; 3 Department of Internal Medicine, Lushuihe Forestry Bureau, Hospital of Jilin Province, Baishan, Jilin Province, China

**Keywords:** Lung adenocarcinoma, Competing endogenous RNA, Long non-coding RNA, Hub, Diagnosis

## Abstract

In our study, we aimed to reveal potential long non-coding RNAs (lncRNA) biomarkers in lung adenocarcinoma (LAD) using lncRNA-mediated competing endogenous RNAs (ceRNAs) network (LMCN). Competing lncRNA-mRNA interactions were identified using the hypergeometric test. Co-expression analysis for the competing lncRNA-mRNA interactions was implemented, and relying on the weight value >0.8, a highly competitive LMCN was further constructed. Degree distribution, betweenness and closeness for LMCN were carried out to analyze the network structure. Functional analyses of mRNAs in LMCN were carried out to further explore the biological functions of lncRNAs. Biclique algorithm was utilized to extract competing modules from the LMCN. Finally, we verified our findings in an independent sample set using qRT-PCR. Based on degrees >60, we identified 4 hubs, including DLEU2, SNHG12, HCP5, and LINC00472. Furthermore, 2 competing modules were identified, and LINC00472 in module 1 functioned as a hub in both LMCN and module. Functional implications of lncRNAs demonstrated that lncRNAs were related to histone modification, negative regulation of cell cycle, neuroactive ligand-receptor interaction, and regulation of actin cytoskeleton. qRT-PCR results demonstrated that lncRNAs LINC00472, and HCP5 were down-regulated in LAD tissues, while the expression level of SNHG12 was up-regulated in LAD tissues. Our study sheds novel light on the roles of lncRNA-related ceRNA network in LAD and facilitates the detection of potential lncRNA biomarkers for LAD diagnosis and treatment. Remarkably, in our study, LINC00472, HCP5, and SNHG12 might be potential biomarkers for LAD management.

## Introduction

Lung adenocarcinoma (LAD) is the most common histological type of lung cancer, which is the leading cause of cancer-related deaths (1). An early and accurate diagnosis may warrant timely treatment to potentially decrease the mortality. However, a critical problem in the progression of LAD is the limited access to early detection and timely treatment. Therefore, understanding the mechanisms underlying LAD progression is urgent for improving the therapy and overall prognosis of this disease. However, traditional recognizable pathological symptoms have limited value in detecting early stage of LAD. Luckily, molecular bio-signatures have been proven to be a promising tool for identifying patients in early-stage disease.

Long non-coding RNAs (lncRNAs), a major class of non-coding (ncRNAs), were determined as ncRNAs with more than 200 nucleotides in length (2). Growing evidence indicates that lncRNAs participate in a wide range of cellular processes, such as genomic imprinting, transcriptional and post-transcriptional regulation (2,3). Significantly, lncRNAs have been implicated to participate in the development and progression of lung cancer. Known lung cancer-associated lncRNAs are few and include MALAT1 (LAD associated transcript) (4), and lncRNA GAS6-AS1 (5). Nevertheless, research about lncRNAs involved in LAD is in its infancy. The identification of LAD-associated lncRNAs and the functions of lncRNAs require further investigation.

Typically, lncRNA functions are characterized using a ‘guilt by association’ strategy (6). It has been reported that lncRNAs harboring miRNA response elements (MREs) serve as competing endogenous RNAs (ceRNAs) to exchange with mRNAs via competing for common miRNAs (7). Experimental studies have demonstrated that aberrant expressions of important lncRNAs of ceRNA network have greater effects on the miRNA-regulated lncRNA/mRNA ceRNA crosstalk interactions, thereby contributing to the occurrence and progression of cancers (7,8). The lncRNA HULC plays an important regulatory role in lung cancer by acting as an endogenous ceRNA (9), revealing the functions of lncRNA-associated ceRNA crosstalk in lung cancer. Further, the dysregulated ceRNA network may provide new hope for exploring the pathogenesis of LAD and detecting new signatures with high accuracy in diagnosis.

In our study, with the goal of better understanding the molecular mechanisms underlying LAD, we aimed to reveal potential prognostic lncRNA biomarkers based on constructing a functional lncRNA-mediated ceRNA network (LMCN) involved in LAD.

## Material and Methods

### Identifying miRNA-target interactions

StarBase v2.0 (http://starbase.sysu.edu.cn/) was developed to systematically detect the RNA-RNA and protein-RNA interaction networks from 108 CLIP-Seq (PAR-CLIP, HITS-CLIP, iCLIP, CLASH) data sets, which provide high-quality experimentally verified miRNA-target interactions manually curated from published studies. In our study, experimentally validated miRNA-mRNA interactions and lncRNA-miRNA interactions were downloaded from Star-Base 2.0.

### Tissue samples and data collection

The mRNA and lncRNA expression data of LAD were recruited from the research of Xi et al. (10) by repurposing the exon-array data on the Affymetrix Human 1.0 ST array from the ArrayExpress database (http://www.ebi.ac.uk/arrayexpress/), which was accessible through E-GEOD-12236. There were 40 samples in E-GEOD-12236, including 20 normal samples and 20 LAD samples. In detail, the probe sets were re-annotated to the human gene symbols, and 17,681 genes were identified. Then, the 17,681 genes were mapped to the miRNA-mRNA interactions and lncRNA-miRNA interactions. Ultimately, expression profiles of 10,485 mRNAs and 57 lncRNAs were identified. Afterwards, we, respectively, extracted the interactions containing any genes of 10,485 mRNAs and 57 lncRNAs from the miRNA-mRNA interactions and lncRNA-miRNA interactions. Totally, 334,014 miRNA-mRNA interactions and 695 lncRNA-miRNA interactions were identified.

### Detecting potential ceRNA interactions

In an attempt to detect competing lncRNA-mRNA interactions, a hypergeometric test was employed in our study, which could evaluate the significance of the common miRNAs between each mRNA and lncRNA. The genome owned K miRNAs, of which M and N were the counts of miRNAs related to the present mRNA and lncRNA, and Y was shared miRNA number of lncRNA and mRNA. With the goal of evaluating the enrichment significance of the shared miRNAs, the P value was calculated using the following equation:

(1) $$\text{P}=1-\sum_{\text{t=0}}^{\text{Y}}\frac{\left(\begin{array}{c} \text{M}\\ \text{t} \end{array}\right)\left(\begin{array}{c} \text{K}-\text{M}\\ \text{N}-\text{t} \end{array}\right)}{\left(\begin{array}{c} K\\ N \end{array}\right)}$$

Then, the original P values were corrected using false discovery rate (FDR) according to Benjamini and Hochberg method (11). A FDR <0.01 was set as the cut-off criterion.

### Construction of a highly competitive LMCN

PCC is an index used to measure the co-expression probability of lncRNA-mRNA pairs. Specifically, PCC was counted according to the expression of the competing lncRNA-mRNAs pairs using the following formula:

(2) $${\text{P}}_{\text{A,B}}=\mathrm{cov}(\text{A, B})/\sigma_{\text{A}}\sigma_{\text{B}}$$

In this formula, cov(A,B) was the covariance of variable A and B, while *σ*A and *σ*B respectively stood for the standard deviations (SD) for A and B. The PCC absolute value of one interaction was defined as the weight value in our work, and only edges with correlations greater than the 0.8 were reserved to construct the LMCN. Cytoscape software (http://cytoscape.org/) was utilized to visualize the highly competitive LMCN.

### Centrality analysis for LMCN

The centrality indexes are widely used for analyzing the properties of network, which contain degree, betweenness, closeness, and eigenvector centrality (12). Among these indexes, the degree is the simplest parameter. As reported, degree is defined as the quantity of links that a node connects with other nodes (13). Betweenness is an index of evaluating the influence of a node exerting over the spread of information through the network. A high betweenness denotes the significant roles of a node in information diffusion (14). Closeness is a measure of the mean length of the shortest paths to access all other proteins in the network (15). The network characteristics of LMCN, including degree centrality, betweenness centrality, and closeness centrality were analyzed using NetworkAnalyzer tool based on log-rank test. In our study, the nodes with degrees >60 were identified as hubs.

### Functional implication of lncRNAs

Database for Annotation, Visualization and Integrated Discovery (DAVID) is a web tool providing a comprehensive set of functional annotation for researchers to understand the biological meaning behind a large number of genes (16). In our study, with the goal of investigating the biological functions of lncRNAs, we conducted gene oncology (GO) and pathway enrichment analysis for genes of LMCN using DAVID based on the ‘guilt by association’ strategy. In detail, we utilized Fisher's exact test to classify the functional terms. Then, P values were adjusted relying on FDR according to Benjamini and Hochberg (11). GO terms with FDR <0.01 were considered statistically significant. Significant pathways were selected relying on FDR <0.05.

### Identifying synergistic, competing lncRNA modules

Since the LMCN can offer a comprehensive view of all possible competing ceRNA interactions, which can be utilized to explore the regulatory characteristics of the lncRNAs, the sub-networks exhibited a more detailed picture of how the lncRNAs synergized with competing mRNAs. Biclique algorithm was recruited from the website of the Computational Biology Laboratory in the Department of Computer Science, Iowa State University (http://genome.cs.iastate.edu/supertree/download/biclique/). Previously, Biclique method was utilized in the study of ceRNA networks (17) to extract competing modules from the LMCN. This synergistic competing module is made up of a complete graph where an edge is realized from every vertex of an lncRNA set to every vertex of an mRNA set. In our study, the Biclique algorithm was employed to extract synergistic competing modules from LMCN.

### Total RNA extraction and qRT-PCR verification

We randomly selected 3 hub lncRNAs in the synergistic, competing lncRNA modules to verify the reliability of our results in LAD patients using qRT-pCR.

A total of 10 LAD patients were included in the current study, after informed consent forms were obtained. Tumor tissues were collected from these LAD patients as the experiment group, while the paired adjacent non-cancerous tissues were also obtained from the LAD patients as the control group. Samples were received with a quality assessment report confirming recruitment of tumor and adjacent non-tumor lung tissues.

Total RNA was isolated with RNA extraction kit (Invitrogen, USA) relying on the manufacturer's protocol. Reverse transcription reaction was implemented by means of A3500 reverse transcription system kit (Promega, USA) according to the manufacturer's protocol. We used the qRT-PCR method to measure the expression levels of candidate lncRNAs according to the real-time PCR system (Applied Biosystems, USA). The qRT-PCR reaction was performed based on the following conditions: 95°C for 30 s, 40 cycles of 95°C for 5 s, and 60°C for 60 s. Each sample was repeated in triplicate. All primers were purchased from Generay Biotech Co., Ltd. (China). The Ct-value for each sample was calculated with the ΔΔCt method (18), and fold change results were shown as 2^-ΔΔCt^. The primer targeting beta-actin mRNA was used as internal control. All data were statistically compared using the paired *t*-test. The statistical analyses were conducted by means of the SPSS 21.0 (IBM, USA).

## Results

### Identification of ceRNA interactions and construction of a highly competitive LMCN

**Figure 1.**
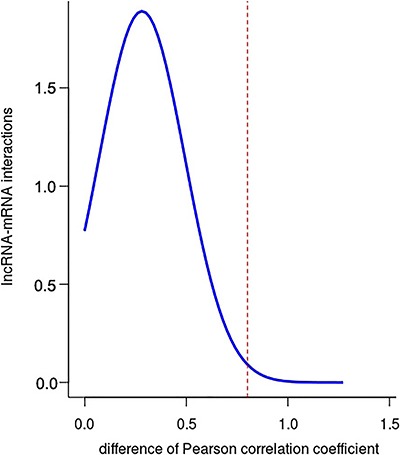
Weight distribution of 18,939 competitive endogenous RNAs (ceRNA) interactions.

**Figure 2.**
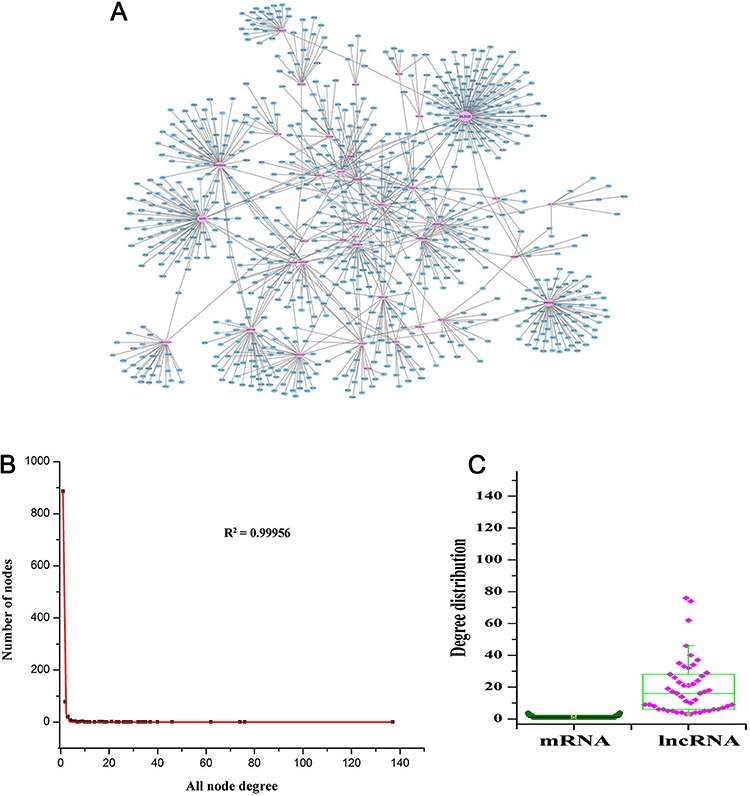
*A*, Highly competitive lncRNA-associated ceRNA network (LMCN). This network contained 55 lncRNAs, 982 mRNAs, and 1104 ceRNA interactions. Pink nodes denote lncRNAs, and blue nodes denote mRNAs. *B*, Degree analysis for the entire network reveals the specific properties of the LMCN. *C*, lncRNA are more critical components relative to mRNA ceRNAs in the LMCN. The lncRNAs had significantly higher degrees than mRNAs in the LMCN (*t*-test, P=2.49E-23).

In order to extract potential lncRNA-mRNA competing pairs, a hypergeometric test was utilized to calculate the significance of the shared miRNAs between each lncRNA-mRNA pair. As a result, a total of 57 lncRNAs, 10,133 mRNAs, and 48,939 ceRNA interactions were selected to establish an original network based on FDR 0.01, and their relationships were not displayed because the data in this original network could not be clearly visualized due to size. Then, PCC was used to measure the co-expression probability of the competing lncRNA-mRNAs pairs, and the absolute value of PCC difference of one interaction in two groups was defined as the weight value. The weight values of all the interactions in the original network are reported in Figure 1. Based on this figure, we found that the weight values ranged from 0.0004 to 1.29. In our study, by applying a weight value >0.8, a high-competing LMCN was established. The LMCN contained 55 lncRNAs, 982 mRNAs, and 1104 ceRNA interactions, and is visualized in Figure 2A. From this figure, we found that the lncRNAs were in the central area of the network, but the mRNAs were typically in the outside layer. We discovered that a large proportion of mRNAs communicated with individual lncRNAs, and lncRNAs served as ceRNAs to connect with multiple mRNAs. These findings demonstrated that the aberrant expression of lncRNA ceRNA might lead to the extensive variation in gene expression through lncRNA-mRNA ceRNA crosstalk interactions, further implicating that ceRNA roles of lncRNAs is crucially important in LAD development and progression.

The original network (57 lncRNAs, 10,133 mRNAs, and 48,939 ceRNA interactions) is shown in Supplementary Table S1, and LMCN (55 lncRNAs, 982 mRNAs, and 1104 ceRNA interactions) is shown in Supplementary Table S2.

### Topological properties of the LMCN

An investigation of the degree distribution of the entire network (R^2^=0.99956) suggested power law distributions (Figure 2B), which demonstrated that the LAD-associated LMCN was a scale-free network. These findings revealed that the LMCN was similar to other biological networks and was well organized by a core set of lncRNA-mRNA competing principles into structured rather than random networks. Generally, a higher degree indicated that the node was a hub, which involved more ceRNA interactions. These results uncovered that although the lncRNAs did not code for proteins, they exhibited more specific degree properties than mRNAs in the LMCN. To extract the hub nodes in the LMCN, all nodes in the LMCN were sorted in a descending order according to their degree distribution. Based on degrees >60, we identified 4 hub lncRNAs, including DLEU2 (degree=137), SNHG12 (degree=76), HCP5 (degree=74), and LINC00472 (degree=62).

**Figure 3.**
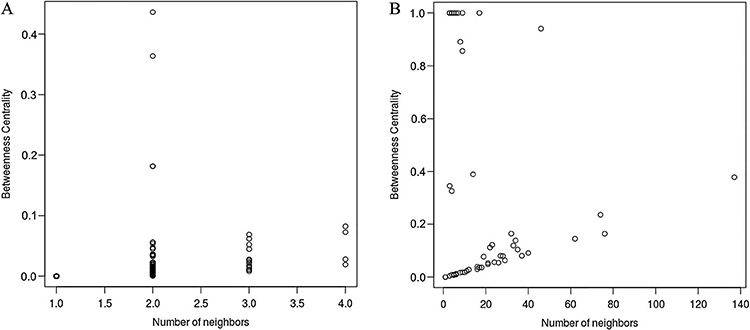
The lncRNA ceRNAs had a higher betweenness centrality than mRNA ceRNAs in the lncRNA-mediated ceRNAs network (LMCN). *A*, Betweenness distribution of mRNAs in the LMCN; *B*, Betweenness distribution of lncRNAs of the LMCN.

**Figure 4.**
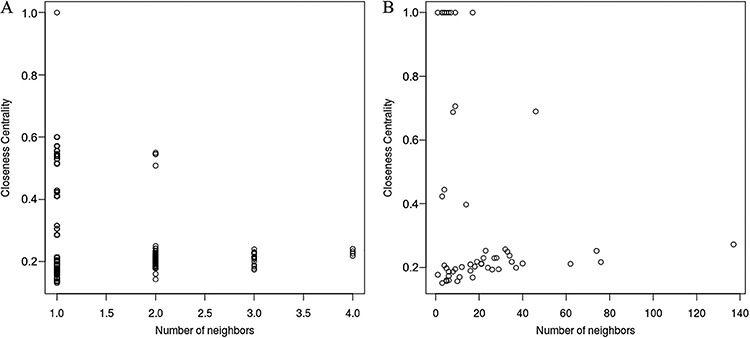
Comparison of closeness between lncRNAs and mRNAs in the lncRNA-mediated ceRNAs network (LMCN). *A*, Closeness distribution of mRNAs in the LMCN; *B*, Closeness distribution of lncRNAs of the LMCN.

Further, comparison analyses on the degree distribution, betweenness centrality, and closeness centrality between mRNAs and lncRNAs were carried out. lncRNAs had higher degree centrality (P=2.49E-23), betweenness centrality, and closeness centrality compared to mRNAs (Figures 2C, 3, and 4), implying that lncRNAs tended to be hub nodes.

### Functional implications of lncRNAs

**Figure 5.**
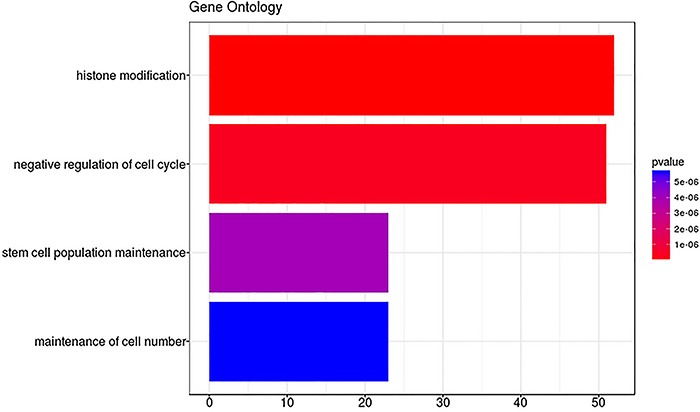
Significant gene oncology terms based on false discovery rate <0.01.

To further verify the potential functional implication of lncRNAs in LAD, we implemented functional enrichment analysis of mRNAs in the LMCN based on GO and KEGG pathways. Based on FDR<0.01, we found that the genes in the LMCN were significantly enriched in four GO terms, including histone modification (FDR=2.25E-04), negative regulation of cell cycle (FDR=1.02E-03), stem cell population maintenance (FDR=6.09E-03), and maintenance of cell number (FDR=6.49E-03). Specific GO terms are shown in Figure 5. Moreover, we observed that the target genes of lncRNAs were remarkably involved in 14 pathways (FDR<0.05), as listed in Table 1. The top 3 pathways were respectively neuroactive ligand-receptor interaction, lysosome, and regulation of actin cytoskeleton. These results demonstrated that lncRNA-associated ceRNA regulation were involved in broad biological functions related to LAD.

**Table 1.** Significant pathway terms based on false discovery rate (FDR) <0.05.

Pathway terms	FDR values
Neuroactive ligand-receptor interaction	0.01404349
Lysosome	0.01404349
Regulation of actin cytoskeleton	0.01404349
p53 signaling pathway	0.01404349
Thyroid hormone signaling pathway	0.01404349
Prostate cancer	0.01404349
Pathways in cancer	0.02321413
Insulin signaling pathway	0.03117087
Acute myeloid leukemia	0.03117087
AMPK signaling pathway	0.04013747
Focal adhesion	0.04013747
B cell receptor signaling pathway	0.04013747
Colorectal cancer	0.04013747
Endometrial cancer	0.04013747

### Identification of highly synergistic, competitive modules

**Figure 6.**
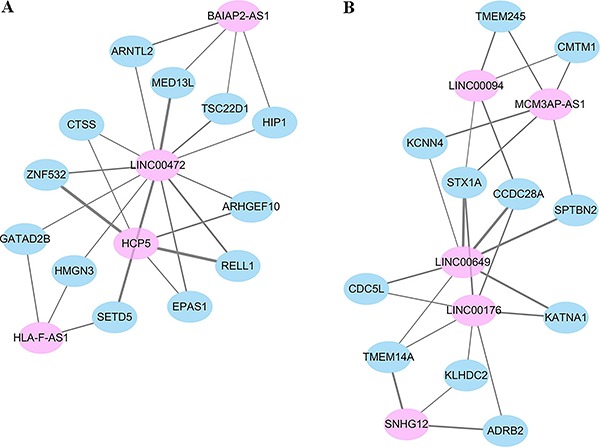
Extraction of synergistic, competing modules. *A*, Module 1. *B*, Module 2. Pink nodes denote lncRNAs, and blue nodes denote mRNAs.

Subsequently, we further investigated the modularity feature of the LMCN. In order to extract synergistic, competing lncRNA modules, the Biclique algorithm was used in this study. In total, 2 synergistic, competitive modules comprising 32 genes were identified, as shown in Figure 6. These modules were numbered 1 and 2. We observed that these modules were the same in size with 16 genes. We also found that in module 1, a hub lncRNA LINC00472 functioned as a hub to compete with 12 mRNAs (GATAD2B, MED13L, TSC22D1, SETD5, HIP1, ARNTL2, HMGN3, EPAS1, RELL1, ARHGEF10, ZNF532, and CTSS) and 3 lncRNAs (HCP5, HLA-F-AS1, and BAIAP2-AS1) in a 16-ceRNA module (Figure 6A), implying its important roles in LAD. These data also indicated that there were synergistic regulatory effects among these 4 lncRNAs. Within the other module (Figure 6B), LINC00649 competed with 7 mRNAs (KCNN4, STX1A, CCDC28A, SPTBN2, CDC5L. TMEM14A, and KATNA1) and 4 lncRNAs (LINC00094, MCM3AP-AS1, LINC00176, and SNHG12).

### qRT-PCR verification

Eventually, we selected 3 hub lncRNAs (LINC00472, HCP5, and SNHG12) in the synergistic, competing lncRNA modules or LMCN to validate the reliability of the above analysis results. LINC00472 competed for many miRNAs, for example, hsa-miR-302e, hsa-miR-520e, hsa-miR-520a-3p, hsa-miR-520b, hsa-miR-520c-3p, hsa-miR-520d-3p, hsa-miR-372-3p, hsa-miR-373-3p, hsa-miR-302d-3p, hsa-miR-302a-3p, hsa-miR-302c-3p, and hsa-miR-302b-3p. Another hub lncRNA HCP5 was found to compete for hundreds of miRNAs, such as hsa-miR-186-5p, hsa-miR-214-3p, hsa-miR-17-5p, hsa-miR-20a-5p, hsa-miR-519d-3p, hsa-miR-653-5p, hsa-miR-93-5p, hsa-miR-106b-5p, hsa-miR-20b-5p, and hsa-miR-106a-5p. Moreover, lncRNA SNHG12 competed for several miRNAs, including hsa-miR-181b-5p, hsa-miR-181a-5p, hsa-miR-16-5p, hsa-miR-15a-5p, hsa-miR-195-5p, hsa-miR-497-5p, hsa-miR-181c-5p, hsa-miR-181d-5p, and hsa-miR-15b-5p. Specific information about miRNAs competed for by lncRNAs are shown in Supplementary Table S3.

**Figure 7.**
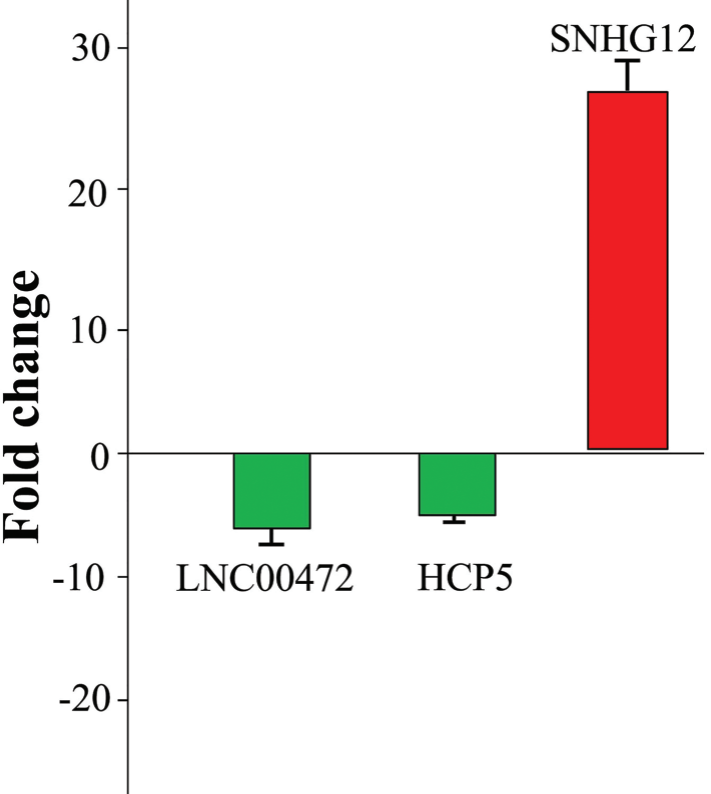
Fold change (2-^ΔΔCt^) of 3 key lncRNAs by qRT-pCR. The primer targeting beta-actin mRNA was used as internal control. Green represents down-regulation, while red represents up-regulation. Data are reported as the mean fold-change±SE of lncRNAs expression in tumor samples relative to normal samples.

The PCR results showed that LINC00472, and HCP5 were downregulated in LAD tissues when compared with adjacent non-tumor lung tissues, while SNHG12 was over-expressed in LAD tissues (Figure 7). The results from the qRT-PCR validation further indicated that candidate lncRNAs including LINC00472, HCP5, and SNHG12 might play important roles in LAD progression, and further verified that our bioinformatics analysis was credible.

## Discussion

Although some methods are available to support LAD diagnosis, for example, biopsy sampling and bronchoscopy, these methods are time-consuming, which may result in delays in early therapy. Thus, to ameliorate this condition, identifying cancer-related genes and the exact mechanisms of LAD occurrence and progression have received increasing attention. As documented, abnormal expression of lncRNAs has been broadly found in many diseases, and aberrant lncRNAs play important roles in biological functions of cancers. The functions of lncRNA are still not well known, and only very few have been well annotated. Hence, as a preliminary exploration for the underlying functional implications of the lncRNAs, ‘guilt by association’ principle was used in our study to reveal biological processes mediated by the lncRNAs biomarkers based on functional enrichment analysis for mRNAs in the LMCN.

In the current study, we observed that mRNAs as ceRNA counterparts of lncRNA signatures were involved in four significant GO terms. Histone modification was the most important GO term in our study. As we all know, histones constantly undergo post-translational modifications, for instance, methylation, acetylation, as well as phosphorylation. Of note, these modifications can modulate the nuclear chromatin setting and is a well-known epigenetic mechanism for regulating gene expression patterns (19). Histone modifications influence various cellular processes including chromatin modification, DNA replication and repair (20,21). Importantly, the modification of histones has been demonstrated to be a predictor for colorectal cancer (22), and gastric carcinomas (23). Moreover, histone H4 modification has been indicated to play an important role in bronchial carcinogenesis and might be a candidate signature for therapeutic approaches in lung cancer (24). Accordingly, the GO term of histone modification might be closely associated with the LAD progression.

Negative regulation of cell cycle was another significant GO term. To our knowledge, the hallmark of cancer is uncontrolled cell proliferation caused by dysregulation of cell-cycle (25). Disturbed surveillance of cell-cycle progression, for example cellular evasion of cell-cycle checkpoints, which are driven by aberrant activation of cell-cycle regulators (cyclins and cyclin-dependent kinases), can cause the tumor development and progression (26). Activated cyclin-dependent kinase-6 and cyclin-dependent kinase-2 during the G1–S cell-cycle transition are key regulators in the modifications of retinoblastoma pathway (27), disruption of which shows strong cell-proliferative activity and has been shown to play important roles in the pathogenesis of lung cancer (28). More importantly, Cai et al. (29) have reported that miR-186 exerts a tumor-suppressive role in the development of LAD, partially through regulation of cell-cycle progression. Hence, it is plausible that the lncRNA biomarkers act as ceRNAs involving the negative regulation of cell cycle, which might exert important functions in the occurrence and progression of LAD.

Based on KEGG pathway analysis, neuroactive ligand-receptor interaction was the most significant pathway in the present study. The effect of a neuroactive steroid reveals a ligand-receptor interaction. Neuroactive steroids have been reported to influence the modulation of GABA receptor, and GABA receptors have been indicated to control cell proliferation (30). Cell proliferation is the hallmark of cancer. Further, growing body of evidence suggests that the actin-based processes, including cell polarity, filopodium formation, and cell migration, might be important in advanced cancers and may be associated with the formation of metastases (31). Thus, our results indicated that the key lncRNAs may exert important functions in initiation and progression of LAD through regulating significant pathways of neuroactive ligand-receptor interaction, and regulation of actin cytoskeleton.

According to the degree distribution, only 1 lncRNA (LINC00472) was a hub node in both LMCN and synergistic module in our study. LINC00472 has also been demonstrated to be abnormally expressed in epithelial ovarian cancer (32). In addition, a former study reported that LINC00472 over-expression significantly decreases the risk of relapse in patients with breast cancer, and LINC00472 also inhibits breast cancer cell proliferation and migration (33). In the current study, the expression level of LINC00472 was decreased in LAD compared to normal group. These results suggested that LINC00472 might play a crucial role in LAD as anti-onco-lncRNA.

HCP5 is named as HLA Class I Histocompatibility Antigen Protein P5, which is localized in the major histocompatibility complex (MHC) class I region. MHC regulates the immune responses relying on the presentation of tumor antigens (34). The regulation of tumor antigen-specific immune responses by an abnormal expression of MHC I molecules has been detected in a variety of cancers (35). Moreover, Liu et al. (36) have indicated that HCP5 is down-regulated in ovarian cancer. Consistent with this, HCP5 was down-regulated in LAD in our study. Thus, we infer that lncRNA HCP5 might be involved in the pathogenesis of LAD.

SNHG12 is a member of small nucleolar RNA host genes (SNHGs), which have been indicated to contribute to cancer progression. Ruan et al. (37) have demonstrated that SNHG12 contributes to cell proliferation and migration. Of note, cell proliferation and migration are the main characteristics of cancer (25). Suppressing SNHG12 can cause the repression of cell proliferation, increased apoptosis, and G1 phase arrest in endometrial cancer (38). Remarkably, this lncRNA SNHG12 has been reported to be over-expressed in colorectal cancer (39) and breast cancer (40). In line with these results, our PCR results exhibited that SNHG12 was up-regulated in LAD, which confirmed that SNHG12 served as an onco-lncRNA in LAD. To the best of our knowledge, we provided the first evidence of SNHG12 dysregulation in LAD. In summary, we believe that over-expression of SNHG12 might promote the progression of LAD.

In conclusion, we constructed a LAD-specific lncRNA-mediated ceRNA network, which enabled an overall view and analysis of lncRNA-associated ceRNA-regulated gene regulation in the progression of LAD on a system-wide level. Our study will help to improve the understanding of lncRNA-mediated ceRNA regulatory mechanisms in LAD progression and provide novel lncRNAs LINC00472, HCP5, and SNHG12 as candidate signatures for diagnosis and therapeutic targets. However, the biological roles of LINC00472, HCP5, and SNHG12 have not yet been determined by animal or patient experiments. Moreover, lncRNAs, miRNAs and mRNAs with differential expressions (between disease and control) might give much more information about potential biomarkers in LAD. In future work, we will construct a dysregulated LMCN to further detect the candidate lncRNA signatures involved in the pathogenesis of LAD.

## Supplementary material

Click here to view [Excel] [pdf].
